# Inflammation, Inflammatory Diseases, and Inflammasomes

**DOI:** 10.3390/ijms24119224

**Published:** 2023-05-25

**Authors:** Young-Su Yi

**Affiliations:** Department of Life Sciences, Kyonggi University, Suwon 16227, Republic of Korea; ysyi@kgu.ac.kr; Tel.: +82-31-249-9644

Inflammation represents the innate immune response of the body tissues against invading microbes and cellular danger signals, and, in this way, it is beneficial [1]. Although inflammation is a body-protective mechanism, chronic inflammation is a key risk factor for developing a variety of human diseases, even cancers [2]. Therefore, many efforts have been made not only on elucidating the molecular and cellular mechanisms of inflammatory responses, but also on developing anti-inflammatory therapeutics. A large number of inflammation studies have mainly focused on the “priming step”, which is the preparation step of inflammatory responses; however, recent studies have focused on another key inflammatory response, the “triggering step”, which is the activation and boosting step of inflammatory responses [3]. The key feature of the triggering step is the activation of inflammasomes, which are intracellular protein complexes comprising pattern recognition receptors and inflammatory molecules [4,5,6,7]. Previous studies have demonstrated the roles of inflammasomes in inflammatory responses and numerous human diseases, providing strong evidence that inflammasomes are the central molecules activating inflammatory responses and are thus new potential targets for novel anti-inflammatory drug development. However, inflammasome functions and their dysregulation in inflammatory responses and human diseases still need to be investigated.

This Special Issue invited original researches, reviews, and perspectives focusing on, but not limited to, the mechanisms of inflammasome regulation, the role of inflammasomes in inflammatory responses and disease, the identification and validation of novel molecules modulating inflammasome functions, and potential inflammasome-targeted therapeutics.

The research article by Cho et al. explored the anti-inflammatory role of Korean red ginseng (KRG) saponins in caspase-11 non-canonical inflammasome-activated inflammatory responses, and the underlying molecular and cellular mechanisms in macrophages. A previous study by the same research group demonstrated that KRG has a strong anti-inflammatory effect by inhibiting caspase-11 non-canonical inflammasome in macrophages [8]. Since ginsenoside saponins are known as key bioactive ingredients in KRG [9], this study further investigated whether the KRG saponin fraction (KRGSF) plays an anti-inflammatory role by inhibiting caspase-11 non-canonical inflammasome in macrophages. KRGSF strongly suppressed the caspase-11 non-canonical inflammasome-activated inflammatory responses by inhibiting the proteolytic activation of caspase-11 and gasdermin D (GSDMD), the GSDMD-mediated pore formation and pyroptosis, and the proteolytic maturation and secretion of pro-inflammatory cytokines, interleukin (IL)-1β, and IL-18 in macrophages [10]. An in vivo study also demonstrated that KRGSF significantly protected the mice from the lipopolysaccharide (LPS)-induced acute lethal sepsis without any significant toxicity [10]. This study provided strong evidence that KRG saponins are critical components that have an anti-inflammatory effect via targeting caspase-11 non-canonical inflammasome in macrophages.

The research article by Kang et al. identified a chemical compound, ODZ10117 (ODZ), and investigated the anti-inflammatory effect of ODZ by inhibiting NLRP3 inflammasome [11]. ODZ specifically inhibited the activation of caspase-1 and NLRP3 inflammasome through inhibiting NLRP3-NEK7 interaction, which is necessary for inflammasome formation [11]. ODZ also significantly decreased the monosodium urate (MSU)-induced IL-1β secretion in the peritonitis mice, and the mortality rate of mice with LPS-induced acute lethal sepsis [11]. This study demonstrated the in vitro and in vivo novel anti-inflammatory effect of ODZ by targeting NLRP3 inflammasome, which suggests that ODZ can be a promising candidate for the treatment of NLRP3 inflammasome-associated immune disorders and cancers.

Another research article by Kanwar et al. undertook a retrospective chart review of 29 consecutive ICU COVID-19 patients receiving dapsone and 30 not receiving dapsone to obtain an indication of whether dapsone can be used as part of the standard treatment of severe COVID-19 in patients entering the ICU [12]. This study found that the neutrophil-to-lymphocyte ratio reduced in 48% of those treated with dapsone and in 30% of those not treated with dapsone, which suggests that dapsone might be lowering the neutrophil-to-lymphocyte ratio [12]. This study also reviewed the collected data on neutrophils being associated with inflammation pathways on which dapsone might act, but did not draw any firm conclusions since this study was not a formal, randomized controlled study of dapsone in severe COVID-19 [12]. However, given the significant decrease in mortality observed in those treated with dapsone and cimetidine, a formal, randomized controlled study of dapsone in severe COVID-19 would be required.

The review article by Xia et al. highlights the studies investigating the post-translational modifications (PTMs) of the components of the NLRP3 inflammasome and the resultant effects on regulating its activity to provide references for the exploration of the molecular mechanisms by which the NLRP3 inflammasome is activated and controlled [13]. This study summarized the regulation of the PTMs, such as ubiquitination, phosphorylation, SUMOylation, alkylation, S-nitrosylation, acetylation, and S-glutathionylation in NLRP3 inflammasome activation [13]. Moreover, this review summarized the dual roles of the PTMs of the NLRP3 inflammasome in the pathogenesis of various cancers, including colitis-associated cancer, colorectal cancer, hepatocellular carcinoma, and melanoma, also showing that it plays a pro-tumorigenic role in breast cancer, colon cancer, colorectal cancer, epithelial skin cancer, fibrosarcoma, and gastric cancer [13]. This review provides new knowledge on the regulation of NLRP3 inflammasome activity by PTMs, thus indicating that the regulation of inflammasome PTMs can represent a new target for the prevention and treatment of inflammasome-associated diseases.

In conclusion, this Special Issue highlights the regulatory roles of inflammasomes and inflammasome-associated molecules in inflammatory responses and diseases and the underlying molecular mechanisms. This study also highlights the critical role of PTMs of the inflammasome components to modulate the functions of the inflammasomes in inflammatory responses and various diseases. We hope that this Special Issue will attract the attention of researchers and urge them to further explore new roles of inflammasomes and inflammasome-associated molecules in inflammatory responses, as these underpin most human diseases. We further hope that this Special Issue will shed light on some of the research conducted to date in relation to the targeting of inflammasomes and inflammasome-associated molecules in the search for novel therapeutics.
